# Parents’ experience of childhood atopic eczema in the public health sector of Gauteng

**DOI:** 10.4102/curationis.v38i1.1215

**Published:** 2015-04-29

**Authors:** Kaarina F. Meintjes, Ann G.W. Nolte

**Affiliations:** 1 Department of Nursing Science, University of Johannesburg, South Africa

## Abstract

**Background:**

The World Allergy Organization found that 20% – 30% of the world's population suffers from an allergic disease. Most allergic patients are seen by non-allergy-trained healthcare workers. The public primary healthcare (PHC) management of childhood atopic eczema (CAE) in the central Gauteng district was the focus of the overall study. The focus of this article is the parents’ experience of CAE and the management thereof. The research question was: What is the experience of parents living with a child with atopic eczema (AE)?

**Objectives:**

The overall purpose was to develop validated PHC management guidelines for CAE. One of the objectives was to explore and describe the experiences of parents regarding the AE of their children and the management thereof.

**Method:**

An embedded single case study design using a qualitative, explorative, descriptive and contextual strategy was employed. Data was collected through semi-structured individual interviews from a purposively selected sample and field notes. Ten parents were interviewed, after which data saturation occurred. Data were analysed according to Tesch's steps of descriptive data analysis. Lincoln and Guba's model was used to ensure trustworthiness.

**Results:**

Three main themes were identified. This article focuses on theme one: The physical, emotional and social impact of CAE. Theme two identified the management challenges and theme three indicated recommendations regarding the management of CAE.

**Conclusion:**

The facilitation of management of CAE focuses on developing PHC guidelines and addressing management challenges in order to achieve better controlled CAE.

## Introduction

The World Allergy Organization (Pawankar *et al.* 2008:S4) found that 20% – 30% of the world's population suffers from allergic diseases and the prevalence is increasing continuously. It is one of the major reasons for people to seek medical help, placing a high burden on health services worldwide. Two South African studies, one in Cape Town and one in Limpopo, indicated a prevalence of atopic eczema (AE) in adolescents at around 19.5% (Wichmann *et al.* 2007:142; Zar, Ehrlich & Weinberg 2004:140). There are no South African statistics available for younger children and no Gauteng statistics at all.

The most common allergic diseases are AE, asthma, allergic rhinitis and allergic conjunctivitis. During the first years of life, eczematous and gastrointestinal symptoms usually predominate in a typical atopic child. Asthma and allergic rhinitis and/or allergic conjunctivitis tend to develop later. This is the so-called ‘atopic march’. AE is not life-threatening, but asthma can be; therefore the development of the atopic march should be prevented (Gray 2011:185; Manjra *et al.* 2005:435).

Eczema is a chronic inflammatory skin disorder that develops mainly in early childhood. There are different types of eczema, of which AE is the most common (Watkins 2010:207). If the child presents with the diagnostic features for atopy, the term ‘atopic eczema’ could be used. The diagnosis of atopy must be confirmed by a positive IgE antibody serum test or positive skin prick tests (Johansson *et al.* 2004:833). These tests are not always available, especially on the primary healthcare (PHC) level. The PHC clinicians (PHCC) should suspect AE if the child presents with three or more of the basic features: flexural lichenification, pruritus, chronic relapse or personal/family history of atopy. The child should also present with three or more of the minor features, namely, xerosis, early age of onset, cutaneous infections, non-specific hand eczema, cheilitis, recurrent conjunctivitis, Dennie-Morgan infraorbital fold, orbital darkening, facial pallor or facial erythema, anterior neck folds, itch when sweating, intolerance to wool and lipid solvents, food intolerance, peri-follicular accentuation, delayed blanch and course of condition influenced by environmental and emotional factors, as classified by Hanifin and Rajka (1980:44–45). The constant itching and scratching that are dominant features of childhood AE (CAE) has a negative impact on the quality of life of both the child and the family (Lewis-Jones 2006:985).

Research showed that AE has a significant effect on the physical and emotional wellbeing of the patient and the family (Camfferman 2011:xviii; Carr 2009:603; Chamlin *et al.* 2004:610; Lewis-Jones 2006:985; Su *et al.* 1997:161; Van Onselen 2009:590). No South African studies have been done on parents’ experience regarding CAE and the management thereof, although the need for research was expressed as early as 2004 (Fisher 2004:68).

### Problem statement

AE is often undertreated, despite the disabling effect it has on the quality of life of the patient (Carr 2009:603; Manjra *et al.* 2005:435). Most allergic patients are managed by non-allergy-trained health workers (Pawankar *et al.* 2008:S4; Potter *et al.* 2009:150). The South African CAE Working Group, a subcommittee of the Allergy Association of South Africa, compiled a consensus document on CAE that provides management guidelines (Manjra *et al.* 2005:436–439), although it does not specifically focus on the PHCCs. The *Standard Treatment Guidelines and Essential Medicines List* (also known as the Essential Drugs List, or EDL) (Department of Health 2008:86–89) is the protocol that needs to be followed by the PHCCs in the public sector in treating patients for various conditions, including AE. The treatment protocol in the EDL gives limited guidance to the PHCCs on the management of CAE. The following research question arose: What is the experience of parents living with a child with AE?

#### Research objectives

The objective of the study was to explore and describe the experiences of parents living with children suffering from AE who were treated in the public sector of the central district of Gauteng.

#### Definition of key concepts

**Atopic:** The World Allergy Organization defines atopy as:

[*a*] personal and/or familial tendency, usually in childhood or adolescence, to become sensitized and produce IgE antibodies in response to ordinary exposures to allergens, usually proteins. As a consequence, these persons can develop typical symptoms of asthma, rhino- conjunctivitis, or eczema. (Johansson *et al.* 2004:833)

In this study, the experience of parents with children with AE was explored.

**Eczema:** Eczema is a chronic inflammatory skin disorder that develops mainly in early childhood but can also develop in adolescence or adulthood (Bieber 2008:1483; Hofer & Leung 2002:327; National Institute for Health and Clinical Excellence 2007:1). This study focused on AE in children and the parents’ experience thereof.

**Childhood:** According to the *Children's Act* No. 38 of 2005 (Department of Health 2005:12), a child is a person under the age of 18. In this study, childhood will refer to children 0–14 years of age.

**Primary healthcare (PHC):** The definition of PHC by the International Conference on Primary Health Care held at Alma-Ata in Russia, 06–12 September 1978 (World Health Organization 1978:1–3) is the departing point for PHC in this study.

PHC in South Africa is the first level of healthcare. According to the African National Congress: National Health Plan for South Africa (African National Congress 1994), PHC forms the central focus for health services. The public health sector of the central district of Gauteng is the context of this study where PHC is mainly rendered at clinics or community health centres by PHCCs.

**Primary healthcare clinician (PHCC):** In the context of this study, a PHCC is a nurse registered with the South African Nursing Council as a general nurse, as well as a community health nurse, who is preferably also registered with the additional qualification in Clinical Nursing Science, Health Assessment, Treatment and Care (South African Nursing Council 1997). This group of PHCCs will be the focus of the study, although medical practitioners who hold a Bachelor in Medicine and Surgery (MBChB) qualification and work full- or part-time in a PHC facility may also be part of the research as they follow the same treatment guidelines as the nurse PHCCs.

**Parent:** ‘A father or mother, one who begets or one who gives birth to or nurtures and raises a child’ (The Free Dictionary n.d.). The parent in this study is the mother or father of a child with AE.

**Experience:** ‘An event or a series of events participated in or lived through’ (The Free Dictionary n.d.). The lived experience of parents with a child suffering from AE is explored.

**Role:** ‘The actions and activities assigned to or required or expected of a person or group’ (The Free Dictionary n.d.). The role of the PHCC in the management of CAE was explored.

#### Contribution to the field

One of the rationales for this study was to provide parents an opportunity to describe how they experience CAE and the management thereof. A conceptual framework regarding the PHC management of CAE was developed, as well as validated PHC management guidelines for CAE. This article reports the parents’ experience of CAE.

## Research design

### Research approach and method

This study was qualitative, explorative and descriptive in nature and the purpose was to gain a new and holistic understanding of the parents’ experience of their child's AE and the management thereof, in the public healthcare sector of the central district of Gauteng.

Semi-structured interviews were held with parents of children aged 0–14 years who suffer from AE. This was part of a single embedded case study. The case in this study was PHC management of CAE in the public sector of in the central district of Gauteng, within the wider context of the public healthcare system. There were three embedded units of this single case and this article focuses on embedded unit one: Parents of children aged 0–14 years of age suffering from AE, who at a stage visited a PHC facility to seek help for their children. There are no statistics available for children suffering from AE in Gauteng.

#### Population and sampling

The public health sector of the central district of Gauteng was the population for this case study. There were three sample groups (embedded units), but as this article focuses on the findings from the parents, only embedded unit one will be discussed.

Sample one (embedded unit one) were the parents of children, aged 0–14 years, who suffer from AE. The inclusion criteria were: the parents must have visited a PHC facility with their child who has AE; they must be able to speak Afrikaans, English, Setswana or Zulu, as these are the languages the skilled interviewer, who holds a Doctorate Curationis in Psychiatry, can speak. These parents were selected purposively as they were information-rich participants of the phenomenon under study. The selection was done as follows: the researcher explained the research to the people in the waiting room; parents who were willing to be interviewed were selected purposively from the waiting room if they met the inclusion criteria; the parents had to give written consent to be interviewed and audio-taped; and the researcher cosigned the consent document.

#### Data collection

Data collection from parents was done using semi-structured individual interviews and field notes. During the interviews, the following were applied to ensure quality data collection:

- The nature and ethical implications of the study were explained in more detail to each participant before starting the interview. The researcher ensured that the participants understood this before giving written consent to be interviewed and audio-taped.
- The skilled interviewer created a relaxed atmosphere by conducting the interview in the language of the participant (English, Afrikaans, Setswana or Zulu), in a conversational manner without interrupting the participant. Care was shown through sensitive and respectful verbal and non-verbal conduct. The researcher took field notes.
- Communication techniques were applied to confirm understanding of what the participant said.
- All interviews were done in a private consultation room.

The following questions were asked of the parents of children, 0–14 years of age, suffering from AE: ‘How is it for you living with a child who suffers from atopic eczema?’ and ‘How do you see the role of PHCCs regarding the management of childhood atopic eczema?’ One pilot interview was conducted to test the question. The pilot interview was conducted with a Zulu-speaking mother of a six-year-old male child who suffers from AE. The question was well understood by the parent and produced rich information. This interview was transcribed and analysed, found to be suitable and was included as part of the data collection and analysis. More semi-structured interviews were conducted. The interviews were conducted in privacy, in a consultation room in the unit. A voice recorder was on the table and the researchers, parent and child were seated around the table. One researcher conducted the interview, whilst the other researcher wrote down the observations during the interview. Toys were provided for children to keep them busy in order for the parent to be able to focus on the interview. Data were collected until data saturation was reached, that is, until no new information emerged.

#### Data treatment

The first step in data analysis was to convert all the evidence into textual form. The interviews were transcribed verbatim. The interviews of parents in languages other than English were transcribed and analysed in the original language by a co-coder who is knowledgeable in qualitative data analysis and who is fluent in Afrikaans, English, Setswana and Zulu. This was done to preserve the original meaning of what was said by the participants. These transcriptions were then translated into English for analysis by the researcher. The data collected through the interviews and field notes were analysed using Tesch's eight steps of the descriptive method of data analysis, as reflected in Creswell (2009:153–160). Themes, categories and subcategories were identified by the researcher and two cocoders, after which consensus discussions were held about the findings. The results are supported by direct quotations of the participants and field notes. The findings were re-contextualised through a literature review.

### Ethical considerations

The Medical Research Council promotes four principles of biomedical ethics, namely, autonomy, beneficence, non-maleficence and justice (Medical Research Council of South Africa 2001). Participants consented voluntarily and were allowed to withdraw at any time in the study without consequence. Information of the participants was confidential and known only to the researcher and skilled interviewers. The raw data are kept under lock and key and will be destroyed two years after publication of the study. The risk to participants involved in this study was negligible. If the parents, of children aged 0–14 years and who were suffering from AE, needed debriefing after the interview, it was provided by the researcher as part of the researcher's professional and ethical responsibility. Ethical clearance was obtained from the University of Johannesburg, Health Science Ethical committee (reference number AEC24/01-2010), as well as the ethical committees of the district authorities in Gauteng (local and provincial authority) and of the tertiary hospital.

### Trustworthiness

The elements for trustworthiness in qualitative research, according to Lincoln and Guba's Model of Trustworthiness (in Krefting 1991:217), are credibility, transferability, dependability and confirmability. These principles were applied throughout the study, as seen in Table 1.

**TABLE 1:** Strategies to ensure trustworthiness.

Strategy	Application
Credibility	Prolonged engagement by spending time: in the unit and primary healthcare facilities with the data during data analysis.
	Open-ended direct observation and keeping of field notes improved the accuracy of the data collected.
	Triangulation: two researchers were involved in the data collection, one conducting the interview and one keeping field notes. The researcher and two cocoders analysed the data.
	Member checking.
	Data analysis was done using the widely recognised Tesch's eight steps of descriptive method of data analysis.
Transferability	A dense description of the research methodology and results.
	Direct quotations of the participants.
	Triangulation as described under credibility.
Dependability	A dense description of the research methodology.
Confirmability	Bracketing and reflexive thinking was applied throughout the data collection and analysis in order to put personal experiences aside.
	Provide a chain of evidence throughout the study: confirmability audit.

## Results and discussion

The findings consisted, firstly, from the demographics of the embedded unit one and, secondly, analysis of the data collected from the semi-structured interviews with parents and field notes.

### Demographics

Ten mothers were interviewed, at which point data saturation occurred. The ages of the children suffering from AE ranged between five months for the youngest child and 12 years eight months for the oldest child. Six of the children were boys and four were girls. Seven of the mothers spoke Zulu, one Setswana and two English. Six of these children were referred from provincial PHC facilities, three from local authority PHC facilities and one was self-referred, but attended a provincial PHC facility initially.

### Themes from the data analysis

Three themes with their categories were identified: theme one – the impact of AE; theme two – management challenges; theme three – recommendations for PHC management of CAE. Theme one with its categories and subcategories is discussed in this article (Table 2).

**TABLE 2:** Results: Theme 1, categories and subcategories: Impact of childhood atopic eczema.

Themes	Categories	Subcategories
1. Impact of childhood atopic eczema	1.1 Physical impact	Extreme physical discomfort
		Extra care and caution needed
		Physically draining to parents
	1.2 Emotional impact	Frustration
		Stressful
		Guilt
		Fear
		Protective
		Satisfaction
		Confusion
		Compassion
		Resilience
		Low self-esteem
	1.3 Social impact	Isolation/low social integration
		Absent school/work
		Support

### Theme 1: Impact of childhood atopic eczema

Theme 1 showed that CAE has a physical, emotional and social impact on both the child and the parent.

#### Physical impact

The physical impact that stood out for the child is the extreme physical discomfort resulting from itching and scratching. This was identified through the interviews as well as direct observation. During the interviews the participants said that the skin looks different (red, rough) and is usually dry and itchy. This causes the children to scratch, often until they bleed. It also came out that the itchiness and scratching interfere with the children's sleep and daily activities. The loss of sleep leads to tiredness and interferes with school work. The skin often becomes secondarily infected because of the constant scratching. Parents said:

‘It used to peel off even if you just touch it, it was just red, the skin was changing to grey, and sometimes it will look like it is rotten.’ (Parent 5)

‘It is on the whole body, even on the face, it is like a crocodile skin.’ (Parent 10)

‘It is very difficult, because he is scratching; he has an amount of blood.’ (Parent 1)

‘At night he feels the pain because it itches this thing, he cries and the worst part is that he is studying and when he is studying he is always scratching himself. It even looks like he is going out of his mind. He will end up sleeping or playing without studying because he is tired.’ (Parent 10)

During the interviews it was observed that all of the children who were in the room had dry skin and were scratching intermittently. One child was very irritable, had extreme dry skin, scratched continuously and had open places on her skin.

The pathophysiology of AE explains the above findings. The barrier function of the skin can be affected by inherited abnormalities, environmental stressors, including irritants, infections and allergens, as well as psychological stress, precipitating AE (Bieber 2008:1485; Cork & Danby 2009:4; Thawer-Esmail 2011:193–197). Both the skin barrier dysfunction and the immune system contribute to IgE-mediated sensitisation and thus AE (Bieber 2008:1485–1492). The main clinical feature of a breakdown in skin barrier is a dry itchy skin, caused by loss of the natural moisturising factor (NMF). This results in the child scratching, causing further disruption of the skin barrier and thus allowing more allergens and irritants to enter the body, causing more disruption of the skin barrier. This forms a cycle, known as the itch–scratch cycle (Cork & Danby 2009:873). The loss of NMF also leads to an increased skin pH level, leading to further breakdown in the skin barrier and delayed recovery thereof, making it easier to develop secondary infections which further aggravate the dysfunctional skin barrier (Cork *et al.* 2009:1892). Therefore, patients with AE are more susceptible to widespread cutaneous viral and bacterial infections (Bieber 2008:1486; Elias 2008:300; Gao *et al.* 2009).

There are different research studies indicating itchiness and scratching, sometimes with bleeding, as the most common physical effect of AE, resulting in a loss of sleep for both the child and the parents (Alanne 2012:55, 65–66; Beattie & Lewis-Jones 2006; Ben-Gashir, Seed & Hay 2004; Camfferman 2011:xviii; Lewis-Jones 2006; Lewis-Jones, Finlay & Dykes 2001; Moore *et al.* 2006:514; Schmitt *et al.* 2011; Su *et al.* 1997:159–161).

The extreme physical discomfort that the child endures is physically draining on the parents, who lose sleep trying to sooth the irritation of the child. One parent said:

‘And at night she is itchy, you are supposed to wake up and help and scratch her at that time, you are also very sleepy, you are just busy scratching until you sleep there and she will wake you up and say, “mama please scratch me” and you see, that is not nice.’ (Parent 5)

Su *et al.* (1997:160–162), Chamlin *et al.* (2004:608–609) and Lewis-Jones (2006:985–986) identified that sleep deprivation led to tiredness and mood changes in parents.

Parents had to do a number of activities differently and exercised extra care and caution because of the special needs of their children with AE. These activities are time-consuming, adding to the physical drain experienced by the parents. These activities included food choices at home and school, ensuring availability of enough treatment, choosing specific clothes, blankets and sheets, cleaning practices at home and hygiene routine for the child, as well as additional involvement at school to help both the child and teacher cope with the illness and schoolwork. Some parents said:

‘I am always cautious … checking the child what he eat.’ (Parent 1)

‘They [*school*] also know that when food is given, they don't give her things like tinned stuff. She takes the allergy medication 30 minutes before the break, and repeats it after 30 minutes so that she should not get affected.’ (Parent 7)

‘There are a lot of things I have to be careful with … whether what I wash her clothes with …’ (Parent 2)

‘He does not even wear his blanket, when the fluffiness of the blanket touches him he gets rash. He has a special sheet that he sleeps with … I place a sheet underneath and then the blanket on top. I make sure that I go to his room every night; you know that a child moves a lot during the night. I wake during the night to check if he is the blanket, even he is not in, I take him back.’ (Parent 9)

‘You know I have to wash every morning her blankets, pyjamas and have to take her mattress outside in the sun and bring in the house at 13h00.’ (Parent 7)

‘You have to choose the clothes. Sometimes it is too hot for her … the wool … you know I have to choose everything … even the hygiene at home … and every time … I have to spring clean because of the dust … I think it also contributes.’ (Parent 2)

‘You will find that there are flies where she sat and I have to always see to it that it is clean.’ (Parent 7)

‘When she started at school, I went to explain to the class teacher.’ (Parent 10)

Chamlin *et al.* (2004:608–609) found that CAE led to more housework, time-consuming treatments, clothing restrictions, altered bath routines, restriction of activities in which the child could participate (such as swimming and outdoor play), less going out for the family, difficulty in getting child care, holding the child's hands to prevent him from scratching and co-sleeping (to prevent scratching and to soothe the child). Lewis-Jones (2006:988) identified additional choice restrictions on the child with AE and the family: holidays, staying with friends and owning pets. These restrictions also restrict a normal family life. This caused parents to put in additional efforts and led to a feeling of physical drainage in the parents. Alanne (2012:53) reported that in families where there was an infant with eczema and food allergies, there was an additional workload (mainly for the mother) related to food choices and preparation of food, informing others regarding how to care for the infant and additional day care arrangements.

#### Emotional impact

To live with children with AE brought out many emotions for the parents.

**Frustration:** This was the emotion that stood out for the parents. They were frustrated, mostly because of the lack of effective and/or sufficient treatment from the PHC facilities. Parents said:

‘What frustrates me most is that … I am given medication and it is not working … *aich* …’ (Parent 1)

‘When you want the medication and I know that the medication helps the child and her condition has worsened and I can't find the medication at [*facility name*]. You will find that they don't give me, what should I do with the child?’ (Parent 5)

This parent sighed a lot during the interview.

The chronic nature of AE and the extreme discomfort of the children aggravated the frustration of parents, as evidenced by the following:

‘The following day maybe, it appears again, what will I say … what will I say …’ (Parent 8)

‘What do I need to give her? Like last night it was terrible – she was crying and scratching till it bleeds and on the hands … I don't know – what should I do?’ (Parent 2)

The limited knowledge of the PHCCs frustrated parents. One parent said:

‘It is difficult, you'll find when it is time to go to the clinic, they do not understand, they see her as if she is positive or whatever, you understand.’ (Parent 7)

Frustration related to the ineffective referral system was also identified. Parents had to wait a long time to be seen by the specialist at the tertiary hospital and had to cope with their child's condition in the meantime:

‘I am very angry with them, because I need them to see her now, because now she is scratching herself!! So, they just gave me for February, but February is very far …’ (Parent 2)

February was still four months away. This parent sighed a lot during the interview and was also very agitated, because no help was immediately available for the child. The interview was stopped to arrange emulsifying ointment from a clinic in a different region to give the parent some relief and to be able to focus on the interview. Thereafter, the interview continued (Field notes).

**Stress:** Parents experienced especially high levels of stress because of the nature of CAE, the management thereof and the understanding of the condition by various role players, including themselves, the PHCCs, friends (playmates, community) and school. Parents said:

‘The itching is not on … she uses her teeth to scratch her hands … her hands are cut …’ (Parent 2)

‘I wish there was a way that it can be treated completely like any other illnesses which is manageable.’ (Parent 5)

‘The school children do not understand and the children who play with him in our street, it was difficult for them, they used to touch her, up until …’ (Parent 7)

‘”Mama, are we passing through”… And I said we are not passing through there, they do not understand what these things are. They see it as if it is something that will infect them.’ (Parent 8)

**Confusion:** Parents experienced confusion regarding the cause for the eczema, applications to the skin, what food to give to the child as well as the fact that sometimes the child will be better and then suddenly the eczema appears again:

‘You are a bit confused to what … makes the reaction, because he can sleep today being better. Tomorrow he wakes up having a lot of rash on the skin and starting to scratch and scratch until blood comes out.’ (Parent 1)

‘I did not know it was eczema … so I was using aqueous cream that is working for anything but it was not good for her.’ (Parent 2)

‘Like sometimes you don't know which food to give, maybe that food is the one that makes the skin to be like that.’ (Parent 3)

**Guilt:** Guilt was experienced by parents because they wondered if they did something that could have caused the eczema. Some parents who also suffered from AE felt guilty because they could have passed it on to the child:

‘You ask yourself what you did wrong that made the child to have eczema. But with me I also had eczema, so it is something that he got from me.’ (Parent 1)

**Fear:** Parents feared the damage that the scratching could cause to the child's body; the possibility that the child might not outgrow the condition; using treatment that was not prescribed by PHCCs; food; and using corporal punishment as the skin is already damaged. The following was said by parents:

‘He also scratches his private parts and I think my child will get hurt and maybe not have children when he is older.’ (Parent 10)

‘I know people say you outgrow it as I have also outgrown eczema, but in this situation of severity, because I can see other people, explaining to the pharmacist that I am also having eczema for thirty-something years! So I become scared sometimes. Is my child going to outgrow this?’ (Parent 1)

‘I never bought it, being scared that these things can burn her and she will change colour, I said to myself let me follow the medication that I get from the clinic.’ (Parent 7)

‘L [*child's name*] is now scared of fish. She thinks that is what causes it.’ (Parent 4)

‘You see, with a child with eczema, you become scared to give her a hiding, because she easily bruises and has marks where you have given a hiding. It does not heal easily; it can take until the following day to heal where you have given him a hiding.’ (Parent 5)

**Protective feelings:** Parents felt protective and acted differently out of concern for their children. Parents would go out of their way to help their children at school and protect them from food or treatment that might be harmful as stated by some parents:

‘I sometimes take a day off at work and go to his school, when the teacher has called me or even when the teacher has not called me, I just go there because I am concerned, it is for the first time that I have a child with this problem.’ (Parent 10)

‘You know sometimes to have a mixture of food … For her; we say at least she must have porridge, you see, so that she must not eat unhealthy food that we eat.’ (Parent 7)

**Satisfaction:** Despite the high level of frustration and stress identified, parents experienced satisfaction when the management of the AE seemed to help. A parent said:

‘What helps me is that I am patient in giving him his medication, because he is becoming better every day. I also see him at times being happy, but when these things start to appear, he is not happy at all.’ (Parent 10)

**Compassion:** Compassion is another emotion identified in the parents. They expressed the pain they felt on behalf of their children and would try and soothe the child:

‘I do understand that eczema is itching … I just rub him, try to sooth the child until he sleeps and just give him all the love and attention that I can.’ (Parent 1)

‘When it is painful, I feel like I should take this thing and put it in me.’ (Parent 4)

**Resilience:** The parents showed resilience regarding the AE of their children. Parents gave priority to the treatment of their children with AE and would do everything within their means to get the children to specialist care and ensure they have treatment. Parents stated:

‘Actually I am amazed, now she comes first … her treatment every day, although it is difficult.’ (Parent 2)

‘Like yesterday, I did not have money; I made means to get it.’ (Parent 5)

‘I was watching TV on Monday on SABC1 … I thought that I will phone … I am going to the Noleen show to ask if she cannot find people that we can start a support group.’ (Parent 7)

Other studies identified the emotional impact that CAE has on both the parent and the child. Parental feelings identified by Chamlin *et al.* (2004:609) were embarrassment about the child's appearance; sadness and depression; guilt and self-blame; restlessness; worry relating to the child's socialisation, self-esteem, cost of care, environmental and/or food allergens, side effects of treatment and the future of the child; dissatisfaction and frustration with the treatment and/or with the physicians; and unsolicited advice from family and friends. A Chinese study identified blame, worry and frustration in the mothers caring for children with AE (Cheung & Lee 2012).

In a South African study done regarding the knowledge, attitudes and practices of Cape Town parents of children with asthma (Jones *et al.* 2000), it was found that they showed resilience and willingness to incorporate environmental measures as part of the management of their child's allergy. In a qualitative Chinese study (Cheung & Lee 2012), one of the findings was that mothers of children with AE constantly looked for different ways to relieve their child's suffering, cure the disease and relieve their own frustrations. Alanne (2012:59) identified that parents would spend additional time and effort regarding the treatment of the child and protecting them against harmful effects of food. A study done by Weber *et al.* (2008) examining the effects of support groups on pruritus and the quality of life of children with AE and their families, found a significant improvement following support group intervention and suggested that this could be a positive non-drug intervention in the management of CAE.

**Low self-esteem:** AE caused physical and emotional trauma in the children as they were irritable, cried a lot and were emotionally labile as a result of the constant itching and scratching. The children are often laughed and stared at and this made them cover their skin. The effects of AE often caused these children to be tired, with impaired concentration, which had an impact on their scholastic achievements and self-esteem. Parents stated:

‘Whenever he wants something I must do it there and then because he is very emotional.’ (Parent 10)

‘She wants to wear clothes that hide everything.’ (Parent 7)

‘But now the teacher told me that he is not coping at all. I must come and sign for the class if he hasn't passed from September 'til December. He just said he will not repeat a class.’ (Parent 9)

Su *et al.* (1997:162) identified behavioural and maladjustment problems as well as lower self-esteem in children with AE. Magin *et al.* (2008) found that the appearance of psoriasis, AE and acne caused intentional or non-intentional bullying by some other children, causing those with AE to be self-conscious. Ben-Gashir *et al.* (2004) found that children with AE felt embarrassed, self-conscious and unhappy as a result of their disease; correlating with the severity of the disease.

#### Social impact

AE leads to isolation and lower social integration of the child and parents as well as missing days at school and work. Support received or the lack thereof also had a social impact.

Parents sometimes caused the isolation of their children by physical and emotional protection of their child with AE:

‘You become more careful and you don't want the child to play with other kids sometimes, just to avoid maybe … either they can get sick from him or maybe my child can get something from other kids as well.’ (Parent 7)

‘She is different you know … I don't want to treat her differently from other kids, but … I know I have to …’ (Parent 2)

Parents seem to be isolated as well:

‘I don't know a person who has eczema.’ (Parent 1)

CAE causes children to miss school as a result of being sick or tired, or because of emotional aspects, such as children laughing at them and a limited understanding of the condition on the part of teachers and school children. Parents said:

‘He will say that he is not going to school because other kids are laughing at him.’ (Parent 10)

‘She said he will infect other children, the kindergarten teacher did not understand, so I used to leave him with neighbours to take care of him.’ (Parent 7)

Parents missed work because their children suffering from AE needed extra care, regular visits to health facilities because of the chronic nature of AE or the unavailability of enough treatment. They said:

‘I could see that this problem was becoming more and more each time. Sometimes I go to the doctor today, then three days later I go back again.’ (Parent 1)

‘When you come back again they will tell you that the treatment is finished or it's not all of it, they give you one and tell you to come back on a certain date … I already took a day off at work; I can't take another day off.’ (Parent 6)

Another social impact of AE expressed by the parents is the support they receive and/or the lack thereof from schools, family and work. Parents said:

‘She said that I must explain to her everything that is happening, how to apply the cream and how I give medication. So I usually give him something to wipe himself first, and the teacher will give cream to apply, the teacher never minded really to do this.’ (Parent 10)

‘Where she used to go to school they did not give her support.’ (Parent 7)

‘He normally stays with the grandmother. She is very supportive and even takes her own medical aid and speak to the doctor … please help the child out.’ (Parent 8)

‘They do understand at work.’ (Parent 6)

Chamlin *et al.* (2004:609–610) identified social subcategories from their study, including adults and other children avoiding interaction with the child with AE. As a result, parents feared the future social integration and social skills development of their children. Chamlin and Chren (2010:285) stated that support groups may help families to develop effective coping strategies, improve treatment compliance and diminish feelings of isolation.

A Canadian study on the burden of AE by Barbeau and Lalonde (2006:35) identified that 1–10 days of work or school was lost as a result of AE and the average absenteeism based on the total population was 1.67 days in 12 months. Fivenson *et al.* (2002) found that 50% of the burden of AE was associated with days lost from work (either absenteeism or loss of productivity).

## Limitations of the study

A single case study design with embedded units was used. If a multiple case study design had been used, cross-validation of findings in other similar contexts would have been possible.

## Recommendations

CAE has a profound physical, emotional and social impact on both the child and the parent. Effective management of CAE is essential for control of the condition, to reduce the number and severity of relapse episodes and to reduce the impact of AE. Three main management challenges for the treatment of the CAE that affected the physical, emotional and social wellbeing, according to the parents, were identified: insufficient or ineffective drug treatment; low knowledge levels of the PHCCs, which impacted on the parents’ knowledge; and an ineffective referral system. Recommendations to address these identified challenges are threefold.

The first recommendation is the development and implementation of PHC clinical guidelines for CAE. This can guide the PHCC to prescribe the correct pharmacological treatment in sufficient quantities and give evidence-based health education regarding AE and its management to the parents. The result of more effective drug and non-drug treatment can help to restore the skin barrier and reduce exposure to allergens and trigger factors. This will lead to less dry and itchy skin, leading to less scratching and skin infections. As the physical wellbeing of the child improves with correct and effective drug and non-drug treatment, so will the emotional and social wellbeing of the child improve. The parents’ physical, emotional and social wellbeing will improve as the AE of their children become more controlled, causing less disruption in their lives.

Secondly, the PHCC's knowledge must improve regarding CAE. The district and subdistrict teams, together with nursing educators, have to provide training opportunities, such as in-service training or workshops. Allergies, including CAE, should be integrated into the nursing curricula.

Thirdly, the district and subdistrict management teams should structure an effective referral system, whereby referred patients could be seen within 2–4 weeks of referral, unless it is an urgent referral such as eczema herpeticum.

## Conclusion

As seen in the discussion of the results, CAE has a physical, emotional and social impact on both the child and the parent. PHCCs must be aware of these effects of CAE and be vigilant in addressing the individual needs and challenges for each child and parent. It is imperative that the PHCC both understand and implement the treatment guideline (which include indications for referral); and keep their knowledge levels updated. If all three recommendations are implemented effectively, the physical wellbeing of both the child and the parent will improve, with a subsequent improvement in their emotional and social wellbeing and an overall better quality of life for the children and their parents.
